# Impact of the COVID-19 Pandemic on Global Diseases and Human Well-Being

**DOI:** 10.3390/jcm11154489

**Published:** 2022-08-01

**Authors:** Arturo Lo Giudice, Maria Giovanna Asmundo, Sebastiano Cimino, Giorgio Ivan Russo

**Affiliations:** Department of Surgery, Urology Section, University of Catania, 95124 Catania, Italy; arturologiudice@gmail.com (A.L.G.); mariagiovannaasmundo@gmail.com (M.G.A.); ciminonello@hotmail.com (S.C.)

## 1. Introduction

This editorial of the Special Issue “Impact of SARS-CoV-2 Pandemic on Global Diseases and Human Well-Being” aims to portray the repercussions of the novel COVID-19 emergency on a wide range of health issues.

The novel acute respiratory syndrome caused by COVID-19 quickly spread after its very first detection on 31 December 2019 in Wuhan, China [1]. Due to the pandemic emergency, many social restrictions were applied and health systems took unprecedented stringent measures that unavoidably influenced people’s lives and disease management. However, according to the study conducted by Kim et al., the incidence of COVID-19 infection is different among various income groups; in detail, these authors analyzed low-, middle- and high-income populations to verify if any increased prevalence of COVID-19 exists in these populations. In this study, an increased possibility of viral exposure was detected among low-income populations, probably due to their living and working environmental conditions, such as poor hygiene, less access to healthcare and crowded living conditions [2]. In conclusion, differences in mortality are reported for people of different income levels in Korea [3].

Since the coronavirus pandemic has undoubtedly impacted every person’s life, it is easy to understand the great effort made to realize a new vaccine. Even though vaccination played a key role in the current emergency scenario, it also poses several problems, including the possibility of side effects that lead to a diffuse rejection of the vaccine by patients [4]. The systematic review conducted by Sessa et al. clarifies that, even though the total rate of severe side effects related to COVID-19 vaccines is very low, it is important to report them in order to advance our knowledge and support our decisions. According to this study and considering the extremely small number of subjects involved in these rare adverse effects (3 to 10 cases per million), it is possible that the thrombotic thrombocytopenia caused by the COVID-19 vaccine may be multifactorial or deeply influenced by genotype; otherwise, several hypotheses exist: it may be caused by the possible cross reactivity of antibodies against the SARS-CoV-2 spike protein with PF4, interactions between spike protein and platelets, the platelet expression of adenoviral proteins and the resulting immune response [5,6].

## 2. Chronic Disease

The COVID-19 pandemic has affected many people worldwide, with serious consequences for many patients. When dealing with the high spread of the novel acute respiratory syndrome caused by coronavirus-19, the most vulnerable patients were considered the most important. Moreover, most patients suffering from chronic diseases such as hypertension, diabetes, chronic kidney disease, and hypercholesterolemia were classified into the high-risk category. In the study conducted by Scicali et al., the impact of the direct and indirect effects of SARS-CoV-2 infection in subjects with familial hypercholesterolemia (FH) is evaluated. Predictably, the percentage of patients affected by FH who consulted lipidologists and/or cardiologists and/or subdued vascular imaging was lower after lockdown compared to the period before, especially because of the fear of contagion. Finally, according to the cohort of 260 patients who took part in the study, the percentage of subjects affected by SARS-CoV-2 was 7.3% and none of them required hospital assistance. Moreover, this study evidenced that the percentage of lipids, through lipid profile evaluation, was lower after lockdown than before (56.5% vs. 100.0%, *p* < 0.01), with a reduction in HDL-C (47.78 ± 10.12 vs. 53.2 ± 10.38 mg/dL, *p* < 0.05), and a relevant increase in non-HDL-C (117.24 ± 18.83 vs. 133.09 ± 19.01 mg/dL, *p* < 0.05). This finding may be explained by the unregulated and sedentary lifestyle that characterized the pandemic period [7]. Moreover, in the pandemic scenario, there has been great interest in the association between SARS-CoV-2 and kidney function; in fact, since the very beginning of the pandemic, several studies analyzed the impact of COVID-19 from different points of view. Precisely, the systematic review and meta-analysis conducted by Chen et al. evaluated the mortality rate, intensive care unit admission, invasive mechanical ventilation, acute kidney injury, kidney replacement therapy and graft loss in the adult kidney transplant population with COVID-19. As is easy to imagine, kidney transplant patients, especially due to their immunocompromised systems, are continually exposed to complications such as opportunistic infections or lymphoproliferative diseases [8]. The higher predisposition and diminished response to infection in the adult kidney transplant population with SARS-CoV-2 disease results in a higher percentage of mortality compared to the general population. In fact, the authors demonstrated increased rates of adverse outcomes among transplanted patients: mortality—21%; admission to intensive care units—26%; intensive mechanical ventilation among those who required admission in intensive care units—72%; acute kidney injury—44%; kidney replacement therapy—12%; and graft loss—8%. Moreover, a higher risk of mortality for elder patients has been registered too [9].

Since the previously mentioned higher risk of mortality and adverse outcomes in patients with chronic diseases is a pressing issue, it is fundamental to assess patients’ increased risk when attending hemodialysis treatments, peritoneal dialysis follow-up or after-transplant visits. In this scenario, the prevalence of SARS-CoV-2 infection among the general population plays a fundamental role in the assessment of the augmented risk of COVID-19 infection among chronic disease patients. As reported in a meta-analysis including 1389 patients, COVID-19 seems to augment the possibility of suffering major consequences among frailer populations. In fact, clinical manifestations of COVID-19 infection are reported to be more serious in aged and pluri-pathological patients. As well as hypertension, diabetes, chronic obstructive pulmonary disease and chronic heart disease, underlying kidney disease seems to be related to a higher incidence of mortality and complications too [10]. In this context, being aware of prevalence and screening test precision will aid doctors in the application of preventative measures to limit COVID-19 spread among more vulnerable populations—such as CKD patients. Unluckily, several factors, such as the sensibility and specificity of screening tests, the type of samples, and the timing of the screening, may alter the final results. However, it is undoubted that to reduce the risks of spread between patients and suffering major consequences, clinicians have to carefully manage the results of the screening test for SARS-CoV-2 [11]. Considering the ever-increasing necessity of an early detection of COVID-19 infection among chronic disease patients, Vial et al. investigated the application of tools routinely used to monitor hemodialysis patients as detection indicators for SARS-CoV-2 disease. In detail, based on a low-cost triage tool, the authors observed that total leukocytes were appreciably lower in patients affected by COVID-19 (4.1 vs. 7.4 G/L, *p* = 0.0072) and were characterized by lower levels of eosinophils (0.01 vs. 0.15 G/L, *p* = 0.0003) and neutrophils (2.7 vs. 5.1 G/L, *p* = 0.021). Moreover, eosinophil count below a certain range (0.045 G/l) seems to be indicative of COVID-19 infection with an AUC of 0.9 [95% CI 0.81–1] (*p* < 0.0001), sensitivity of 82%, specificity of 86%, a positive predictive value of 82%, a negative predictive value of 86% and a likelihood ratio of 6.04. In conclusion, these results suggest the possibility of the early detection ofSARS-CoV-2 by a cheap and easily accessible tool such as CBC [12].

## 3. Everyday Life

The COVID-19 pandemic has affected modern society both from a strictly health perspective and from a social perspective, regarding everyday life implications. Of course, some people in particular situations have been affected more than others, and our editorial is focused on the stress perceived by the caregivers of patients with Alzheimer’s disease (AD) during the pandemic.

The “caregiver burden” consists of the emotional, physical, social, or financial burden that the caregiver feels in caring for his/her family member. The caregivers’ perceptions of stress can be influenced by psychosocial factors, such as kinship, cultural and social aspects [13,14].

A study has been published in our editorial that evaluates the psychological responses of caregivers of individuals with dementia during the COVID-19 pandemic lockdown; a cross-sectional survey using an anonymous online questionnaire was used [15].

The questionnaire included three sections that presented closed-ended questions with five-point Likert scales and binary-type questions (except for the first one, which collected socio-demographic data). This survey consisted of (1) caregivers’ sociodemographic data (gender, age, education, residential position in the last 14 days, marital status, working status, and type of relationship with the patient being assisted) and information about the patient’s illness; (2) psychological scales to assess the impact of the COVID-19 pandemic; and (3) tools investigating caregivers’ physical and mental health.

Eighty-four AD patients’ caregivers were involved in this study by answering an online questionnaire. The data showed that caregivers were affected by high burden and stress; in fact, they obtained a high mean score on the Perceived Stress Scale. Moreover, caregivers’ burden was mainly related to their patients’ physical difficulties (assessed by Caregiver Burden Inventory—Physical Burden) and perception of losing time (assessed by Caregiver Burden Inventory—Time-dependence Burden). Moreover, caregivers perceived their quality of life as very low (assessed by Short Form-12 Health Survey Physical and Short Form-12 Health Survey Mental Health). Finally, this study demonstrated that participants mostly used dysfunctional coping strategies, such as avoidance strategies (assessed by Coping Orientation to Problem Experiences—Avoidance Strategies); however, these approaches did not affect their stress levels.

## 4. Mental Health

The deterioration of sociopsychological status and mental health due to governmental restrictions after the spread of COVID-19 has been widely investigated in our Special Issue, with one study aiming to clarify who, when, how, and why individuals died by suicide in Japan during the COVID-19 pandemic. This study assessed a change in the percentage of suicide during the pandemic compared to the pre-pandemic period using a governmental database that divided subjects by prefectures, gender, age, means, motive, and household factors using a linear mixed-effects model [16].

Suicide mortality decreased during the first stay-home order and increased after the first stay-home order ended. Furthermore, the direct health hazard of COVID-19 itself functioned as a suicide suppressor; nevertheless, the protraction of the COVID-19 pandemic period deeply contributed to the increasing incidence of suicide, especially for females. Contrary to nationwide fluctuation patterns, the suicide mortality incidence in metropolitan regions for both genders, male and female, did not decrease during the first stay-home order. Other factors, such as gender (female), age (adolescents), one-person household residents, and living in metropolitan areas, were possible risks of increasing suicide mortality in 2020. The reduction in SMR-S in all 47 prefectures during the first stay-home order might be compared to the “honeymoon period” phenomenon. The stabilization of suicide mortality observed during each stay-home order may also suggest people becoming accustomed to the pandemic.

## 5. Sexual Health

### Pre-COVID

An Italian study carried out between 1 June and 31 December 2019, involving people of any gender and sexual orientation, aimed to describe the most common kinds of contemporary sexual behaviors in Italy prior to the COVID-19 pandemic. Participants were recruited via social media posts on Facebook and Instagram, and Google Forms was used to create and deliver the survey online. Each of the 12,590 people who took part in this study consented to fill out the survey. The survey questions assessed a range of factors, including the frequency and pleasure of various sexual activities (self-stimulation, being masturbated by a partner, masturbating one’s partner, receiving and providing oral sex, vaginal penetration, receiving and providing anal penetration), sexual satisfaction, the frequency of orgasms, triggers for auto-eroticism, the use of sex toys, the pleasantness of various sexual fantasies, pornography use, betrayal, traumatic sexual experiences, stress, contraception, protection against sexually transmitted infections, the use of medications or drugs, the use of dating apps or sites and sexting. Most participants were heterosexual, 10,153 (80.6%), followed by homosexuals (234), bisexuals (2087; 16.6%), and pansexuals (83; 0.7%). Only 20–30% of participants in the poll used sex toys, while the majority watched pornography on a weekly basis (27.8 %) and alone (80%). Having intercourse in public, having sex with multiple people at once, having sex while blindfolded, being tied up, and watching a naked person are the fantasies that most stimulate and excite the participants. About 80–90% of the respondents indicated that they did not engage in anal intercourse; it is probable that, in Italy, sexual independence and the urge to test out novel sexual practices may be eclipsed by a widespread sense of shame [17].

It was shown that crises can alienate loved ones; moreover, the loss of a home can alter daily habits and couples’ sex lives. The repercussions of the lockdown on the Italian population’s sex lives are less known. In Italy, crises that have changed peoples’ habits (e.g., the L’Aquila earthquake) have been widely studied. After this earthquake, a high rate of sexual dysfunction-related symptoms was reported among young adults, particularly in subjects experiencing post-traumatic stress disorder [18]. The lockdown period has likely led to changes in Italians’ sex lives. Precisely, in this Special Issue, we aimed to investigate if any change in adult men and women’s sexual behaviors occurred during lockdown.

An Italian, multicenter, cross-sectional study, was conducted in 15 urological centers. This research was performed through a Google Forms online survey, from 4 May 2020 (50 days after the start of the lockdown) to 18 May 2020. Inclusion criteria were sexually active subjects in stable relationships for at least 6 months; any age and gender were included. Exclusion criteria were subjects who were affected by COVID-19, single or sexually inactive. In the end, 2149 participants were enrolled in this study. The results showed that 29% of subjects considered that their sex lives with their partners had “much or very much” deteriorated during the lockdown period; otherwise, 49% considered it to be “much or very much” improved during the same period. Finally, 225 did not report any deterioration or improvement.

Among participants who reported an improvement in their sex lives with their partners, the greatest percentage was represented by women; this result was found to be significantly associated with cohabitation, having a stable relationship for more than 5 years and being married without children. No patients of any gender reported having sexual dysfunction. On the other hand, most of the participants who reported a worsening of their sex lives with their partners did not live with their partners during lockdown (73.4%). Among cohabitees, most had sons (82%) and a stable relationship for more than 5 years (81.7%). Among the women that reported a worsening of their sex lives with their partners, there were no sexual disfunction but a higher level of anxiety, tension, fear, and insomnia; on the other hand, men who reported worsening of their sex lives with their partners had a higher rate of mild erectile dysfunctions, orgasmic dysfunctions, and low sexual satisfaction within the previous 4 weeks.

However, despite the impossibility of meeting friends and relatives during lockdown, a reconciliation took place between cohabiting couples. Most people expressed satisfaction having their partners at home. The improvement was reported primarily in participants who had been in stable relationships for more than 5 years, probably because the increased time spent together favored the rediscovery of a feeling that the couple might have lost in their life routines. Moreover, spending entire days at home can stimulate and facilitate common interests between partners—the sharing of hobbies or daily practices that normally could not be shared because of a lack of time. Participants over the age of 40 improved more than those under the age of 40, probably because most younger participants did not live with their partners during that time [19].
